# Chemogenomic Profiling of Antileishmanial Efficacy and Resistance in the Related Kinetoplastid Parasite Trypanosoma brucei

**DOI:** 10.1128/AAC.00795-19

**Published:** 2019-07-25

**Authors:** Clare F. Collett, Carl Kitson, Nicola Baker, Heather B. Steele-Stallard, Marie-Victoire Santrot, Sebastian Hutchinson, David Horn, Sam Alsford

**Affiliations:** a London School of Hygiene and Tropical Medicine, London, United Kingdom; b Wellcome Trust Centre for Anti-Infectives Research, School of Life Sciences, University of Dundee, Dundee, United Kingdom

**Keywords:** *Leishmania*, *Trypanosoma*, amphotericin B, aquaglyceroporin, major facilitator superfamily transporter, miltefosine, paromomycin, phospholipid-transporting ATPase, sodium stibogluconate, vesicle-associated membrane protein

## Abstract

The arsenal of drugs used to treat leishmaniasis, caused by *Leishmania* spp., is limited and beset by toxicity and emergent resistance. Furthermore, our understanding of drug mode of action and potential routes to resistance is limited. Forward genetic approaches have revolutionized our understanding of drug mode of action in the related kinetoplastid parasite Trypanosoma brucei. Therefore, we screened our genome-scale T. brucei RNA interference (RNAi) library against the current antileishmanial drugs sodium stibogluconate (antimonial), paromomycin, miltefosine, and amphotericin B. Identification of T. brucei orthologues of the known *Leishmania* antimonial and miltefosine plasma membrane transporters effectively validated our approach, while a cohort of 42 novel drug efficacy determinants provides new insights and serves as a resource. Follow-up analyses revealed the antimonial selectivity of the aquaglyceroporin TbAQP3. A lysosomal major facilitator superfamily transporter contributes to paromomycin-aminoglycoside efficacy. The vesicle-associated membrane protein TbVAMP7B and a flippase contribute to amphotericin B and miltefosine action and are potential cross-resistance determinants. Finally, multiple phospholipid-transporting flippases, including the T. brucei orthologue of the *Leishmania* miltefosine transporter, a putative β-subunit/CDC50 cofactor, and additional membrane-associated hits, affect amphotericin B efficacy, providing new insights into mechanisms of drug uptake and action. The findings from this orthology-based chemogenomic profiling approach substantially advance our understanding of antileishmanial drug action and potential resistance mechanisms and should facilitate the development of improved therapies as well as surveillance for drug-resistant parasites.

## Abstract (precis)

The arsenal of drugs used to treat leishmaniasis, caused by *Leishmania* spp., is limited and beset by toxicity and emergent resistance. Furthermore, our understanding of drug mode of action and potential routes to resistance is limited. Forward genetic approaches have revolutionized our understanding of drug mode of action in the related kinetoplastid parasite Trypanosoma brucei.

## INTRODUCTION

The kinetoplastid parasites *Leishmania* species, Trypanosoma brucei subspecies, and Trypanosoma cruzi are endemic throughout much of the tropics and subtropics, sub-Saharan Africa, and Latin America, respectively. They are responsible for various forms of leishmaniasis (*Leishmania* spp.) (1), human African trypanosomiasis (HAT) (T. brucei *gambiense* and T. brucei *rhodesiense*), the livestock disease nagana (T. brucei *brucei* and related African trypanosomes) (2), and Chagas’ disease (T. cruzi) (3). Collectively, these parasites cause a huge burden of disease among predominantly poor populations in affected regions. Leishmaniasis is caused by a range of *Leishmania* species, leading to cutaneous and visceral forms of the disease, of which there are 0.7 million to 1.3 million and 0.2 million to 0.4 million cases per year, respectively (4). While cutaneous leishmaniasis can be self-limiting, infections with Leishmania braziliensis (and other members of the *Viannia* subgenus) can develop into mucocutaneous leishmaniasis, a profoundly disfiguring form of the disease (4). Visceral leishmaniasis (VL), also known as kala-azar, is typically fatal if untreated.

There are four current antileishmanial drugs, sodium stibogluconate (SSG), paromomycin, miltefosine, and amphotericin B, which are unsatisfactory due to toxicity, emerging drug resistance, complex administration protocols, and variable efficacy depending on the disease type or infecting *Leishmania* species (5). With the exception of miltefosine (in use against leishmaniasis since 2002), the current antileishmanial drugs have been in use for many decades. Until recently, efforts have focused on the development of more-effective drug delivery regimens and combination therapies, with the aim of reducing dosages (and, therefore, side effects) and combating the emergence of resistance. The rise of antimonial-resistant Leishmania donovani on the Indian subcontinent now precludes the use of SSG (6), while miltefosine-resistant L. donovani has been confirmed in the clinic (7). Consequently, the World Health Organization recommends various combination therapies, depending on the *Leishmania* species and geographical region (8). However, it is relatively easy to generate *Leishmania* parasites resistant to combination therapies in the laboratory (9, 10). More recently, new drugs have entered the clinical development pipeline. However, the most advanced of these, fexinidazole, which recently passed phase 2/3 clinical trials against HAT (11) and has antileishmanial activity *in vitro* (12), lacks efficacy *in vivo* (13).

Given the ease with which *Leishmania* parasites become resistant to the available drugs, it is critically important to understand how this resistance might develop. Identification of the genetic changes underlying drug resistance will enable the development of molecular diagnostics to inform treatment choice (14). *Leishmania* genome and transcriptome analyses have identified large numbers of candidate genes (15, 16), but relatively few have been directly linked to drug action. While some drugs can freely move across membranes, many are taken up via specific surface receptors and transporters. For example, miltefosine uptake is dependent on a *Leishmania* amino phospholipid-transporting (P4)-type ATPase (or flippase) and its β-subunit/CDC50 cofactor, Ros3 (17, 18), while the Sb(III) form of SSG is taken up via an aquaglyceroporin, AQP1 (19). There is also evidence that the ABC transporter MRPA influences SSG uptake and sequestration (20), and several other proteins have been implicated in SSG efficacy (reviewed in reference 14). In addition, the generation of drug-resistant *Leishmania* in the laboratory and various omics analyses have provided insights into antileishmanial drug action and resistance mechanisms. Proteomic analyses of paromomycin-resistant L. donovani revealed a complex picture, with a range of proteins being upregulated, including several involved in translation regulation, vesicular trafficking, and glycolysis (21). A similar analysis of amphotericin B-resistant Leishmania infantum highlighted the differential expression of metabolic enzymes and the upregulation of proteins involved in protection against reactive oxygen species (22). Metabolomic analyses suggested that oxidative defense also contributes to SSG-amphotericin B and SSG-paromomycin resistance in L. donovani (23).

The studies described above highlight the phenotypic consequences of changes in drug sensitivity but not necessarily the genetic changes responsible. Forward genetic approaches can identify genes that contribute to drug action and resistance. For example, genome-scale RNA interference (RNAi) library screening, coupled with RNA interference target sequencing (RIT-seq), has revolutionized our understanding of anti-HAT drug action and resistance (24, 25). In addition, cosmid sequencing (Cos-seq) has enabled gain-of-function screening in *Leishmania* (26), leading to target validation for *N*-myristoyltransferase (27) and the identification of a panel of putative antimony and miltefosine resistance genes (28). While undoubtedly a powerful technique, Cos-seq is unable to identify drug uptake or activation mechanisms, which can be characterized by loss-of-function approaches such as RIT-seq. However, due to the absence of the RNAi machinery in most *Leishmania* species (with the notable exception of *L. braziliensis* [29]), this loss-of-function approach is not possible for these parasites. Although T. brucei and *Leishmania* have distinct life cycles, they are phylogenetically related kinetoplastid parasites that exhibit a high degree of biochemical and genetic similarity (30). Indeed, the majority of orthologous genes are syntenic, indicating little change in gene order since divergence from a common ancestor. Perhaps not surprisingly, then, several “dual-purpose” drugs display activity against both parasites, including pentamidine (5), fexinidazole (11, 12), and the proteasome inhibitor GNF6702 (31). T. brucei is also susceptible to *in vitro* killing by the four current antileishmanial drugs. Therefore, we hypothesized that T. brucei RNAi library selection with the antileishmanial drugs would enable the identification of candidate drug efficacy determinants with orthologues in *Leishmania*.

Here, we describe RIT-seq library screening using each of the current antileishmanial drugs. We identified 44 high-confidence putative drug efficacy determinants, including T. brucei orthologues of the *Leishmania* SSG and miltefosine transporters (MTs). Among many previously unknown drug efficacy determinants, we found that the vesicle-associated membrane protein TbVAMP7B contributes to miltefosine and amphotericin B efficacy and highlight a role for a cohort of amino phospholipid-transporting P4-type ATPases (or “flippases”) in driving amphotericin B efficacy. This collection of validated and putative antileishmanial drug efficacy determinants provides new insight into mode of action and potential resistance mechanisms and represents an important resource to guide future study.

## RESULTS

### Orthology-based chemogenomic profiles for antileishmanial drugs.

**FIG 1:**
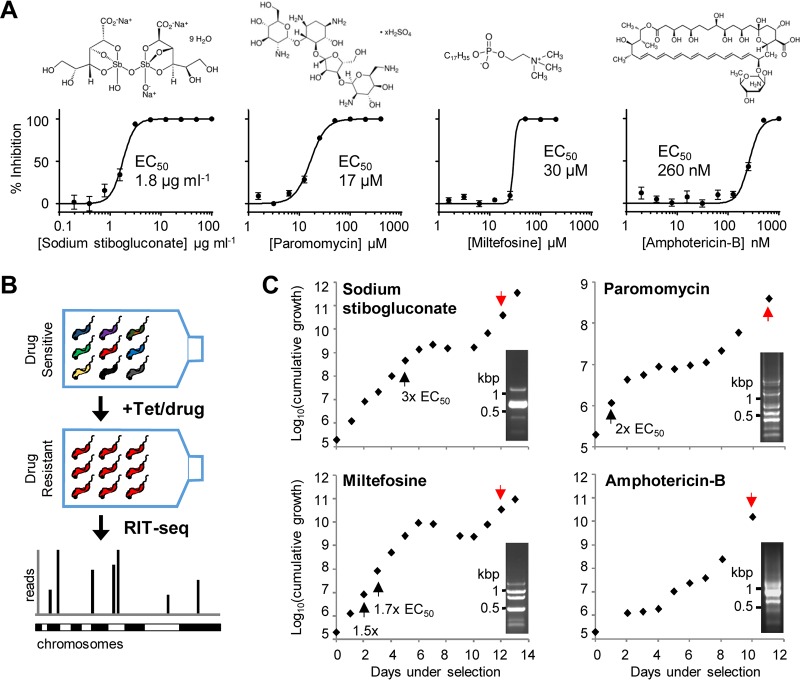
Antileishmanial drug selection of a genome-scale T. brucei RNAi library. (A) Representative EC_50_ charts showing the susceptibility of T. brucei to the antileishmanial drugs. Individual EC_50_ assays were carried out in quadruplicate; error bars represent standard deviations. Insets show structures of the antileishmanial drugs (downloaded from https://en.wikipedia.org/wiki/Sodium_stibogluconate and Sigma-Aldrich). (B) Schematic showing bloodstream-form T. brucei RNAi library selection and RNAi fragment identification by RIT-seq. Tet, tetracycline. (C) Growth during antileishmanial drug selection of the BSF T. brucei RNAi library. Selection was initiated at 1.5× EC_50_, except for miltefosine (1.0× EC_50_), and adjusted as indicated (black arrows); induction with 1 μg · ml^−1^ tetracycline was maintained throughout. Genomic DNA was prepared at the indicated times (red arrows). Insets show RNAi library-specific PCR.

The four current antileishmanial drugs SSG, paromomycin, miltefosine, and amphotericin B have *in vitro* 50% effective concentration (EC_50_) values against T. brucei of 1.8 μg · ml^−1^, 17 μM, 30 μM, and 260 nM, respectively (Fig. 1A). The equivalent values versus intracellular L. donovani amastigotes in mouse peritoneal macrophages are approximately an order of magnitude higher (SSG and paromomycin) or lower (miltefosine and amphotericin B) (32). To identify factors whose loss renders T. brucei less sensitive to each antileishmanial drug, a bloodstream-form (BSF) T. brucei RNAi library was induced for 24 h, and each drug was then added at 1× to 3× EC_50_; selection and induction were maintained thereafter (Fig. 1B). After selection for approximately 10 days, populations with reduced drug sensitivity emerged and grew consistently under continued selection (Fig. 1C).

**FIG 2:**
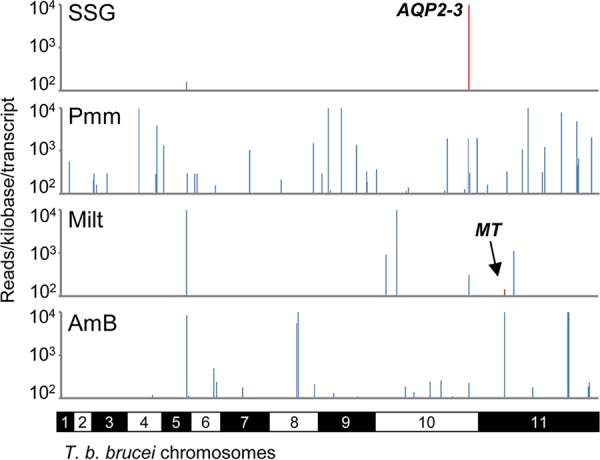
Genome-scale maps showing hits in each screen. Illumina sequencing of the amplified RNAi target fragments identifies T. brucei orthologues of known *Leishmania* drug transporters and novel putative drug efficacy determinants. RNAi fragments amplified from each selective screen were mapped against the TREU927 T. brucei reference genome. Red bars correspond to T. brucei orthologues of known *Leishmania* drug transporters: *AQP2-3* (aquaglyceroporin-2-3 locus) (Tb927.10.14160-70) and *MT* (miltefosine transporter orthologue) (Tb927.11.3350). The *y* axes are truncated to 10^4^ reads/kb/transcript. SSG, sodium stibogluconate; Pmm, paromomycin; Milt, miltefosine; AmB, amphotericin B.

Following robust growth for at least 2 days, genomic DNA was isolated from the drug-selected populations and subjected to RNAi construct-specific PCR, generating distinct banding patterns for each (Fig. 1C). We sequenced the amplified RNAi target fragment populations from the selected RNAi libraries on an Illumina HiSeq platform (see Table S1 in the supplemental material). For each selected RNAi library, we mapped more than 3 million individual sequence reads, representing antileishmanial enriched RNAi target fragments, to the TREU927 T. brucei reference genome (33) using our established RIT-seq methodology (34) (Fig. 1B). The presence of the RNAi construct-specific barcode identified “high-confidence” hits, i.e., those represented by more than 99 barcoded reads/kb/predicted transcript (open reading frames plus predicted untranslated regions, as annotated in the TREU927 reference genome available at www.tritrypdb.org) and recovery of at least two independent RNAi target fragments (Fig. 2, Fig. S1, and Table S1).

Importantly, we identified T. brucei orthologues of two known *Leishmania* determinants of antileishmanial drug efficacy. RNAi target fragments that mapped to the *TbAQP2-3* locus (Tb927.10.14160-70), which encodes two aquaglyceroporins, dominated the SSG-selected RNAi library; L. donovani AQP1 (LdBPK_310030.1) is a key mediator of SSG uptake (19). Another significant hit identified following miltefosine selection was a putative flippase (Tb927.11.3350); the corresponding coding sequence is syntenic with the L. donovani miltefosine transporter (LdBPK_131590.1) (17). The identification of T. brucei orthologues of these known antileishmanial efficacy determinants highlights the power of this chemogenomic profiling approach in the identification of mechanisms of action and resistance that are also relevant to *Leishmania* parasites. In addition to these hits, our RIT-seq analyses yielded a further 42 high-confidence hits (Fig. 2, Fig. S1, and Table S1).

### TbAQP3, an orthologue of *Leishmania* AQP1, is linked to antimonial action.

Aquaglyceroporin defects in T. brucei and in *Leishmania* have been linked to arsenical and antimonial resistance (see above), but specific relationships among drugs and AQPs have not been fully elucidated. For example, TbAQP2 is responsible for pentamidine and melarsoprol uptake (35), possibly via receptor-mediated endocytosis in the former case (36), and mutations that disrupt *TbAQP2* are responsible for melarsoprol resistance in patients (37). L. donovani AQP1 has also been linked to antimonial resistance in patients (38). Notably, TbAQP3 and *Leishmania* AQP1 have the same set of selectivity filter residues (NPA/NPA/WGYR), while TbAQP2 has a divergent set (NSA/NPS/IVLL) (39). Therefore, we investigated the specificity of the interaction between SSG and TbAQP2/TbAQP3, the major hits in the SSG screen.

**FIG 3:**
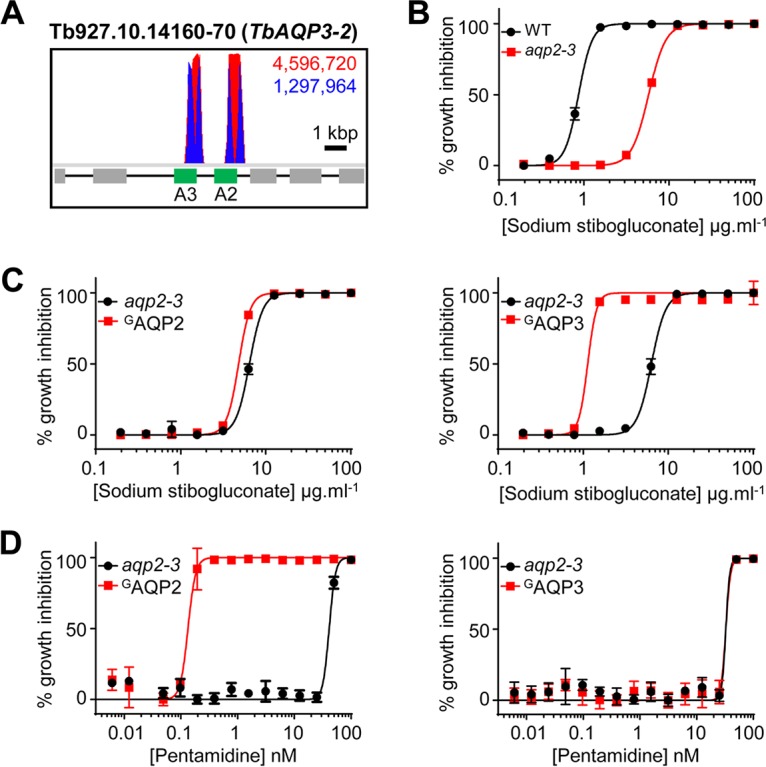
TbAQP3, a T. brucei orthologue of *Leishmania* AQP1, is selective for sodium stibogluconate. (A) Total (red) and RNAi construct-specific 14-mer-containing (blue) reads mapping to the *TbAQP2-3* locus, *Tb927.10.14160-70*. Targeted open reading frames are highlighted in green; flanking open reading frames are in gray. (B) Sodium stibogluconate EC_50_ assay following deletion of the T. brucei *AQP2-3* locus (*aqp2-3*). (C and D) Sodium stibogluconate (C) and pentamidine (D) EC_50_ assays following expression of ^GFP^AQP2 (^G^AQP2) (left) and ^GFP^AQP3 (right) in *aqp2-3*-null T. brucei. Individual EC_50_ assays were carried out in quadruplicate. Error bars represent standard deviations. WT, T. brucei wild type for the *AQP2-3* locus.

Sequence mapping of the RNAi target fragments following SSG selection revealed that approximately 71% and 29% of mapped reads containing the RNAi construct-specific barcode corresponded to *TbAQP2* (Tb927.10.14170) and *TbAQP3* (Tb927.10.14160), respectively (Fig. 3A); only 0.08% of reads mapped elsewhere in the genome. These data are consistent with the idea that both aquaglyceroporins contribute to SSG action. However, the *TbAQP2* and *TbAQP3* coding sequences are 82.3% identical; thus, while an RNAi fragment may unambiguously map to *TbAQP2*, it may be sufficiently similar to *TbAQP3* to elicit its depletion. Therefore, we tested the relative contribution of the encoded aquaglyceroporins to SSG action against T. brucei using *aqp2-3*-null and reexpression cell lines (35).

Deletion of the *TbAQP2-3* locus led to a 6.7-fold increase in the SSG EC_50_ (Fig. 3B), consistent with the output from the screen. Inducible expression of green fluorescent protein-tagged TbAQP2 (^GFP^TbAQP2) in the null cell line had little effect on T. brucei SSG sensitivity (Fig. 3C, left); however, ^GFP^TbAQP3 expression reduced the SSG EC_50_ 5.5-fold (Fig. 3C, right). In contrast, and as shown previously (35), ^GFP^AQP2 expression complemented the pentamidine resistance of *aqp2-3*-null T. brucei (Fig. 3D, left), while ^GFP^AQP3 expression had no effect on pentamidine sensitivity (Fig. 3C, right). Therefore, SSG sensitivity and resistance are specifically determined by TbAQP3 expression. This indicates that the NPA/NPA/WGYR selectivity filter, present in both TbAQP3 (39) and *Leishmania* AQP1, may be selective for antimonial uptake.

### T. brucei lysosomal MFST influences aminoglycoside action.

**FIG 4:**
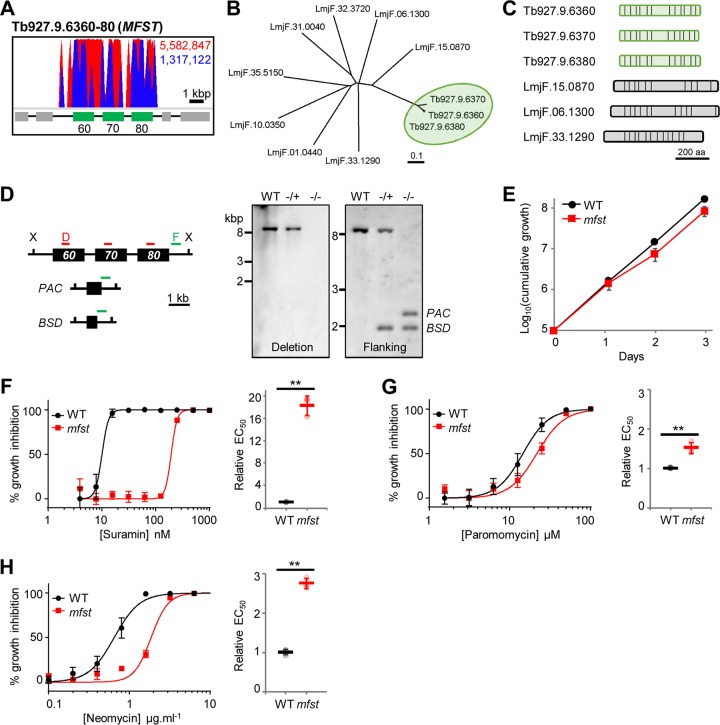
The T. brucei lysosomal major facilitator superfamily protein influences the efficacy of aminoglycoside drugs. (A) Total (red) and RNAi construct-specific 14-mer-containing (blue) reads mapping to the *MFST* locus, *Tb927.9.6360-80*. Targeted open reading frames are highlighted in green; flanking open reading frames are in gray. (B) Unrooted neighbor-joining tree comparing representative *Leishmania* MFST proteins with Tb927.9.6360-80 (highlighted in green) (see Fig. S3 in the supplemental material for an extended tree). (C) Predicted *trans*-membrane organization of the Tb927.9.6360-80 proteins and the selected *Leishmania* proteins (vertical bars, TM domains). aa, amino acids. (D) *MFST* locus deletion strategy and Southern hybridization confirming the generation of heterozygous (−/+) and homozygous (−/−) *MFST* locus-null T. brucei parasites. X, XhoI; D, deletion probe; F, flanking probe; *PAC*, puromycin acetyltransferase; *BSD*, blasticidin S deaminase; WT, wild type. (E) Growth of WT and *MFST* locus-null (*mfst*) T. brucei parasites in culture. (F to H) Representative data from EC_50_ assays comparing the sensitivities of WT and *mfst* T. brucei parasites to suramin (F), paromomycin (G), and neomycin (H). Inset charts summarize EC_50_ data from three independent biological replicates. Individual growth (E) and EC_50_ (F to H) assays were carried out in triplicate and quadruplicate, respectively. Error bars represent standard deviations. *P* values were derived from Student’s *t* test (**, *P* < 0.01).

Selection of the BSF T. brucei RNAi library with the antileishmanial aminoglycoside paromomycin identified 50 hits, 28 of which fulfilled our high-stringency criteria (Table S1). Twenty-one of the high-confidence hits were functionally annotated and included several associated with transport and nucleic acid processing. The top three hits with functional annotations were *Tb927.9.6360-80* (major facilitator superfamily [MFS] transporters [MFSTs]), *Tb927.11.6680* (amino acid transporter [AAT15]), and *Tb927.11.14190* (Tudor domain-containing staphylococcal nuclease [TSN]) (40), targeted by approximately 84%, 1.7%, and 0.9% of the mapped reads, respectively (Fig. 4A, Fig. S2, and Table S1). However, while parasites able to deplete AAT15 and TSN persisted in the population over the 12 days of selection with paromomycin, we were unable to detect a significant advantage versus wild-type T. brucei during the course of a standard 72-h EC_50_ assay (Fig. S1). Therefore, we focused our attention on the MFST genes.

The genes at the *Tb927.9.6360-80* locus share at least 92% sequence identity and encode three putative MFSTs, a ubiquitous family of proteins responsible for membrane transit of a wide range of solutes, including drugs (41). Comparison with the sequences annotated “MFS” or “major facilitator superfamily transporter” in the L. major reference genome confirmed that the syntenic coding sequence, *LmjF.15.0870*, is most closely related to *Tb927.9.6360-80* (Fig. 4B and Fig. S3). The *Leishmania* and T. brucei proteins share similar *trans*-membrane (TM) domain organizations and the cytoplasmic loop between TM6 and TM7, which is characteristic of MFST proteins (Fig. 4C) (42).

We previously identified the *Tb927.9.6360-80* locus as a key contributor to suramin efficacy against T. brucei, with RNAi depletion of the three transcripts leading to a 10-fold reduction in parasite sensitivity to suramin; localization studies also indicated that at least one of these transporters is lysosomal (24). Deletion of the whole locus (Fig. 4D) revealed that the three encoded proteins are collectively dispensable in cultured BSF T. brucei (Fig. 4E) and enabled us to confirm that these proteins influence not only suramin efficacy (Fig. 4F) but also those of paromomycin (Fig. 4G) and the related aminoglycoside neomycin (Fig. 4H). While loss of these MFST proteins dramatically reduces suramin efficacy, the effect on paromomycin and neomycin sensitivity is less pronounced (1.5- and 2.8-fold EC_50_ increases, respectively) although significant. Our mutant BSF T. brucei parasites also exhibited better tolerance than wild-type parasites to the aminoglycosides at concentrations equivalent to or greater than the EC_99_ during the first 24 h of exposure (Fig. S4).

### TbVAMP7B, a cross-efficacy determinant for amphotericin B and miltefosine.

**FIG 5:**
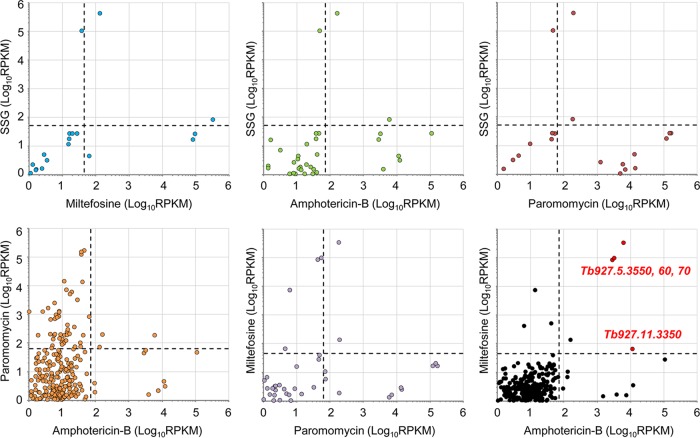
Pairwise comparisons identify putative amphotericin B-miltefosine cross-efficacy loci. Shown are pairwise comparisons of the sequenced outputs from the four selective screens. Data were converted to reads per kilobase per million mapped reads (RPKM) to control for minor interlibrary variations in read depth. Dashed lines represent stringent 100-read cutoffs for each selected RNAi library converted to RPKM. High-confidence cross-efficacy determinants following comparison of the miltefosine- and amphotericin B-selected RNAi libraries are highlighted in red in the top right quadrant.

To identify antileishmanial cross-efficacy determinants, we next used pairwise comparisons of RNAi library screen outputs (Fig. 5). We first identified a small cohort of hits represented by at least two RNAi target fragments and >99 reads/kb/transcript in more than one screen. This group included the *AQP2-3* locus, represented by at least 100 reads in all four screens. We did not explore this locus further since the read count was at least 3 orders of magnitude lower in each screen than in the SSG screen, and leishmanial AQPs have not been implicated in resistance to the other drugs (see above). Two other loci fulfilled our stringency criteria, and both were enriched following amphotericin B and miltefosine selection, *Tb927.5.3550-70* and *Tb927.11.3350* (Table S1); further analysis of the former hit is considered in this section, while the contribution of Tb927.11.3350 to drug action is addressed subsequently.

**FIG 6:**
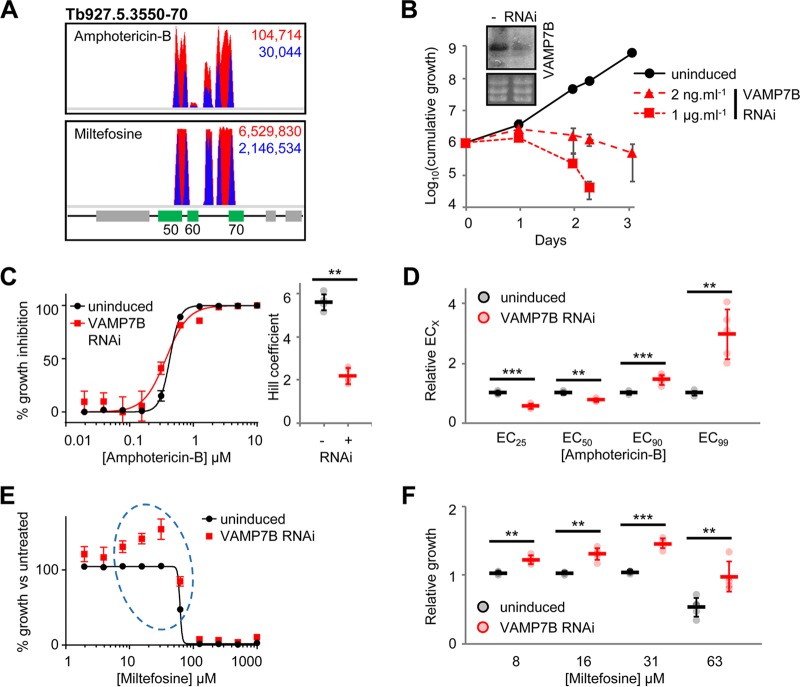
T. brucei VAMP7B, Tb927.5.3560, and the action of amphotericin B and miltefosine. (A) Total (red) and RNAi construct-specific 14-mer-containing (blue) reads mapping to *Tb927.5.3550-70* following amphotericin B and miltefosine selection. Targeted open reading frames are highlighted in green; flanking open reading frames are in gray. (B) T. brucei population growth following TbVAMP7B (Tb927.5.3560) RNAi knockdown. The inset shows confirmation of RNAi knockdown by Northern blotting following 24-h induction with 1 μg · ml^−1^ tetracycline; an ethidium bromide-stained gel is shown as a loading control. (C) Representative data from a 30-h amphotericin B EC_50_ assay following TbVAMP7B RNAi knockdown induced with 2 ng · ml^−1^ tetracycline. The inset chart summarizes Hill coefficient data for five biological replicates. (D) Effect of TbVAMP7B RNAi knockdown on EC*_X_* for five biological replicates. Data for each replicate were derived from EC_50_ values and Hill coefficients presented in panel C. (E) Representative data from a 30-h miltefosine EC_50_ assay following TbVAMP7B RNAi knockdown induced with 2 ng · ml^−1^ tetracycline. Data are plotted to show population growth relative to untreated T. brucei (uninduced or induced). The dashed ellipse highlights miltefosine-mediated complementation of the Tb927.5.3560 RNAi growth defect. (F) Chart summarizing T. brucei population growth in the presence or absence of TbVAMP7B RNAi at a subset of miltefosine concentrations from five independent biological replicates. Individual growth (B) and EC_50_ (C and E) assays were carried out in triplicate and quadruplicate, respectively. Error bars represent standard deviations. *P* values were derived from paired Student’s *t* test (**, *P* < 0.01; ***, *P* < 0.001).

RIT-seq analysis revealed that 2.2% and 97% of mapped reads identified *Tb927.5.3550-70* in the amphotericin B and miltefosine screens, respectively (Fig. 6A). This locus encodes a thioredoxin-like protein (*Tb927.5.3550*); a vesicle-associated membrane protein, TbVAMP7B (*Tb927.5.3560*) (43); and a hypothetical protein (*Tb927.5.3570*). Analysis of the RNAi target fragments mapping to *Tb927.5.3550-70* revealed that few uniquely targeted the *TbVAMP7B* coding sequence (Fig. 6A). Instead, the RNAi target fragments that mapped to the flanking genes overlapped either the *TbVAMP7B* coding sequence (*Tb927.5.3550* RNAi target fragments) or the 3′ untranslated region (*Tb927.5.3570* RNAi target fragments). This pattern is consistent with the poor tolerance of TbVAMP7B depletion. Our previous high-throughput phenotypic analysis indicated that TbVAMP7B RNAi knockdown is associated with a significant loss of fitness, while depletion of the flanking transcripts had a less dramatic effect (Table S1) (44). Taken together, these data suggested that TbVAMP7B is an amphotericin B-miltefosine cross-efficacy determinant, while the identification of the flanking genes was due to bystander effects.

To test this hypothesis, we generated stem-loop RNAi BSF T. brucei cell lines targeting TbVAMP7B and Tb927.5.3570. As predicted, depletion of Tb927.5.3570 had no effect on growth or sensitivity to amphotericin B or miltefosine (Fig. S5). In contrast, knockdown of TbVAMP7B following induction with tetracycline at 2 ng or 1 μg · ml^−1^ resulted in a significant growth defect (Fig. 6B). To assess the contribution of TbVAMP7B to drug efficacy, we induced RNAi with 2 ng · ml^−1^ tetracycline for 24 h and assessed drug sensitivity over a further 30 h under inducing conditions. Incubation with low-concentration tetracycline and a shorter EC_50_ analysis (as opposed to the standard 72-h protocol) ensured that the growth defect due to TbVAMP7B RNAi knockdown was minimized while still allowing us to test the protein’s contribution to drug action.

Unexpectedly, RNAi knockdown of TbVAMP7B reduced the amphotericin B EC_50_, by 24% (Fig. 6C). However, TbVAMP7B depletion also resulted in a significant decrease in the Hill coefficient. Consequently, while the EC_50_ decreased upon TbVAMP7B depletion, the EC_90_ and EC_99_ increased 1.45-fold and 3-fold, respectively (Fig. 6D); the EC_25_ decreased by 44%, consistent with the effect on the EC_50_ and the change in the Hill coefficient. Therefore, small changes in TbVAMP7B expression can lead to a significant loss of sensitivity to high-concentration amphotericin B while enhancing sensitivity to the drug at a low concentration. This relative resistance to high-concentration amphotericin B explains the enrichment of TbVAMP7B-targeting RNAi fragments following selection of the RNAi library at 1.5× EC_50_. In contrast, miltefosine at relatively low concentrations complemented the TbVAMP7B RNAi growth defect and further increased growth at lower concentrations (Fig. 6E and F).

Our findings indicate specific interactions between TbVAMP7B and both amphotericin B and miltefosine. VAMP7 proteins are involved in endosome and lysosome membrane fusion (45), and it is notable in this respect that amphotericin B disrupts membranes and that miltefosine is a phospholipid drug. TbVAMP7B depletion does not significantly increase the EC_50_ for either drug, but nevertheless, these interactions may be important in a clinical setting where exposure will be variable in different tissues and at different times following dosing.

### Multiple hits link amphotericin B action to phospholipid transport and metabolism.

**FIG 7:**
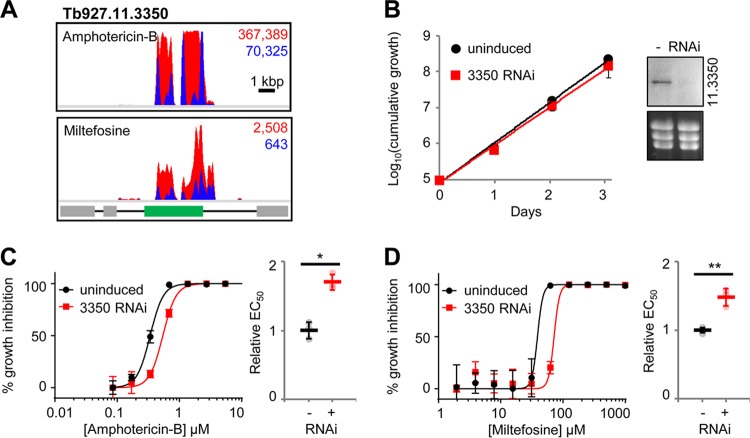
The T. brucei miltefosine transporter orthologue, Tb927.11.3350, influences miltefosine and amphotericin B efficacy against T. brucei. (A) Total (red) and RNAi construct-specific 14-mer-containing (blue) reads mapping to *Tb927.11.3350* following amphotericin B (AmB) or miltefosine selection. Targeted open reading frames are highlighted in green; flanking open reading frames are in gray. (B) T. brucei population growth following RNAi knockdown of Tb927.11.3350. The inset shows confirmation of RNAi knockdown by Northern blotting; an ethidium bromide-stained gel is shown as a loading control. (C and D) Representative data from amphotericin B and miltefosine EC_50_ assays following RNAi knockdown of Tb927.11.3350. Inset charts summarize data from three independent biological replicates. Individual growth (B) and EC_50_ (C and D) assays were carried out in triplicate and quadruplicate, respectively. Error bars represent standard deviations. *P* values were derived from Student’s *t* test (*, *P* < 0.05; **, *P* < 0.01). RNAi inductions were carried out with 1 μg · ml^−1^ tetracycline.

Our amphotericin B screen yielded 13 high-confidence hits, for which Gene Ontology (GO) term profiling revealed links to membranes and lipids (Table S2 and Fig. S6). This is consistent with disruption of membranes by amphotericin B. Miltefosine uptake in *Leishmania* is dependent on a flippase (17, 18), which also contributes to the antileishmanial action of amphotericin B (46). RNAi fragments targeting the syntenic locus in T. brucei, *Tb927.11.3350*, were enriched following selection with amphotericin B and miltefosine (Fig. 5 and Fig. 7A). Depletion of Tb927.11.3350, while having no effect on parasite growth in culture (Fig. 7B), led to a reproducible increase in amphotericin B and miltefosine EC_50_s (Fig. 7C and D). RNAi knockdown also significantly enhanced short-term survival in high-concentration amphotericin B and miltefosine (Fig. S6). Therefore, as in *Leishmania*, the T. brucei miltefosine transporter orthologue contributes to the action of miltefosine and amphotericin B.

**FIG 8:**
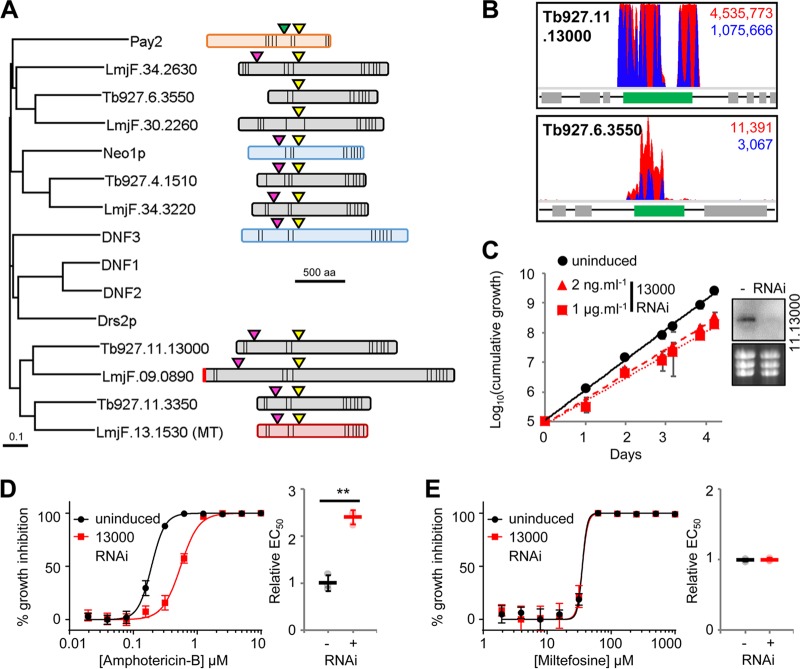
Flippases influence the action of amphotericin B. (A) Neighbor-joining phylogenetic tree showing the T. brucei and L. major flippases versus the S. cerevisiae flippases (Neo1p [UniProt accession number P40527], Drs2p [accession number P39524], and DNF1-3 [accession numbers P32660, Q12675, and Q12674]) and a representative cation-transporting P-type ATPase (Pay2 [E9P982]). Shown are schematics of predicted T. brucei and L. major flippases, including the miltefosine transporter (MT) (red), and representative S. cerevisiae flippases (Neo1p and DNF3) (blue) and P-type ATPase (Pay2) (orange). Conserved domains (actuators, TGES [green triangles] and DEGT [pink triangles]; phosphorylation, DKTGT [yellow triangles]), predicted signal peptides (vertical red bar), and predicted *trans*-membrane domains (vertical black bars) are highlighted. (B) Total (red) and RNAi construct-specific 14-mer-containing (blue) reads mapping to *Tb927.11.13000* and *Tb927.6.3550* following amphotericin B selection. Targeted open reading frames are highlighted in green; flanking open reading frames are in gray. (C) T. brucei population growth following RNAi knockdown of Tb927.11.13000. The inset shows confirmation of RNAi knockdown by Northern blotting; an ethidium bromide-stained gel is shown as a loading control. (D and E) Representative data from amphotericin B and miltefosine EC_50_ assays following RNAi knockdown of Tb927.11.13000. Inset charts summarize data from three independent biological replicates. Individual growth (C) and EC_50_ (D and E) assays were carried out in triplicate and quadruplicate, respectively. Error bars represent standard deviations. *P* values were derived from Student’s *t* test (*, *P* < 0.05; **, *P* < 0.01). RNAi inductions were carried out with 1 μg · ml^−1^ tetracycline, unless otherwise stated.

In addition to Tb927.11.3350, the T. brucei genome contains three other putative flippases (Fig. 8A) as well as three putative β-subunits, including Tb927.11.13770, the syntenic orthologue of *Leishmania* Ros3 (18). Three of the four flippases (Tb927.4.1510, Tb927.11.3350, and Tb927.11.13000) have a domain organization similar to that of the Saccharomyces cerevisiae flippases and possess the DEGT and DKTGT motifs characteristic of the actuator and phosphorylation domains (47). The fourth, Tb927.6.3550, lacks the flippase DEGT domain, although it clusters with the *Leishmania* flippase LmjF.34.2630. However, it also lacks the TGES domain characteristic of related cation-transporting P-type ATPases such as yeast Pay2 (47), so its identity is unclear (Fig. 8A).

In addition to the *Leishmania* miltefosine transporter orthologue, Tb927.11.3350, RNAi fragments targeting the flippases Tb927.11.13000 and Tb927.6.3550 and the β-subunit Tb927.11.13200 were enriched following selection with amphotericin B, with Tb927.11.13000 represented by 78% of mapped reads (Fig. 8B and Table S1). Targeted RNAi depletion of Tb927.11.13000 led to a mild growth defect (Fig. 8C) and a >2-fold EC_50_ increase, validating this protein as an amphotericin B efficacy determinant in T. brucei (Fig. 8D). The impact of Tb927.11.13000 depletion was most pronounced during the initial 24 h of drug exposure, enabling the parasite population to increase approximately 1.3-fold and 4-fold over 8 and 24 h, respectively, in the presence of 0.7 μM (>EC_99_) amphotericin B (Fig. S6). The uninduced population declined by more than 40% and 60% over the same periods. In addition, while exposure to 1.8 μM (>EC_99.9_) amphotericin B led to an 80% decline in the induced population over 24 h, cultures of uninduced cells were cleared within 4 h of exposure to this drug concentration (Fig. S6). Depletion of this putative phospholipid-transporting ATPase had no effect on miltefosine efficacy (Fig. 8E), confirming its specific contribution to amphotericin B action.

Our results reveal that multiple T. brucei flippases drive the efficacy of amphotericin B, all of which have syntenic orthologues in *Leishmania* (Fig. 8A). Therefore, in addition to the well-characterized miltefosine-transporting flippase, other *Leishmania* flippases may play significant, and potentially specific, roles in the antileishmanial action of amphotericin B and miltefosine.

## DISCUSSION

**FIG 9:**
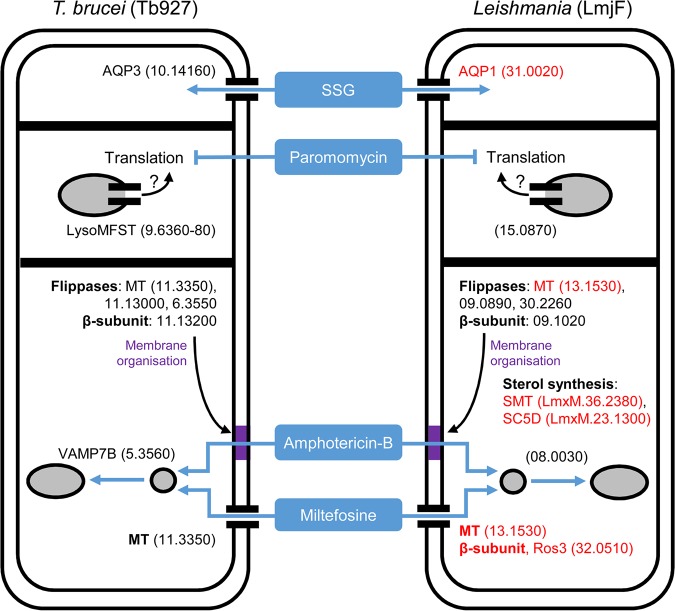
Known and candidate drivers of antileishmanial drug efficacy in *Leishmania*. The key T. brucei proteins identified in our antileishmanial loss-of-function screen (left) and their *Leishmania* orthologues (right) represent candidate antileishmanial drug efficacy determinants. Red denotes known *Leishmania* drivers of antileishmanial efficacy whose loss of function reduces drug efficacy (see the text for details). The strain prefix for the truncated gene identifications is at the top of each panel, with the exception of the sterol biosynthetic enzymes recently shown to contribute to amphotericin B efficacy against L. mexicana (73). Gray-filled circles (endosomes) and ellipses (lysosome) represent the endocytic system. The purple blocks represent membranes modified by changes in sterol biosynthesis and the putative action of the flippases and their β-subunit; changes in membrane composition anywhere in the endocytic system may influence the intracellular transit of amphotericin B or its ability to form ion-permeable channels. Tb927.11.3350 has an intracellular localization in procyclic-stage T. brucei; however, in the current absence of data from BSF T. brucei, we speculate that this protein localizes to the plasma membrane in BSF T. brucei, as per its *Leishmania* orthologue.

In the current absence of an effective genome-scale loss-of-function screen in *Leishmania*, we speculated that selection of a T. brucei RNAi library would provide insights into antileishmanial drug action while also revealing novel T. brucei biology. By selecting our genome-scale BSF T. brucei RNAi library with the current antileishmanial drugs followed by RIT-seq analysis, we identified a panel of putative antileishmanial drug efficacy determinants (see Table S1 and Fig. S1 in the supplemental material). SSG and miltefosine selection identified TbAQP3, an orthologue of the known Sb(III) transporter, and Tb927.11.3350, the T. brucei orthologue of the *Leishmania* miltefosine transporter (MT), respectively, confirming the power of this approach. In addition to these known drug transporters, we validated several novel drug efficacy determinants identified by our selective screens: Tb927.9.6360-80 (paromomycin), Tb927.5.3560 (miltefosine and amphotericin B), and Tb927.11.13000 (amphotericin B). Our results highlight the role of a lysosomal transporter in paromomycin efficacy, emphasize the importance of membrane composition for the action of amphotericin B and miltefosine, provide insight into the substrate selectivity of the trypanosomatid aquaglyceroporins, and present several new candidate antileishmanial drug efficacy determinants (Fig. 9).

SSG contains Sb(V), which is not reduced in *Leishmania* medium, limiting its efficacy against the extracellular promastigote stage (48). However, once it enters the host macrophage, it is thought to be reduced to the toxic Sb(III) form, which can be taken up by intracellular *Leishmania* amastigotes via AQP1 (19, 49). In contrast, we speculate that Sb(V) is reduced to Sb(III) in T. brucei HMI9 medium due to the presence of supplementary l-cysteine (50). Thus, in common with intracellular *Leishmania* amastigotes, cultured extracellular T. brucei parasites treated with SSG are exposed to toxic Sb(III). T. brucei RNAi library selection with SSG and our subsequent validation experiments identified a single efficacy determinant, TbAQP3. Aquaglyceroporins are ubiquitous transporters of water, glycerol, and other small solutes, whose specificity is defined by their selectivity filter residues. *Leishmania* AQP1 and the T. brucei proteins TbAQP1 and TbAQP3 have the same selectivity filter, NPA/NPA/WGYR, while TbAQP2 possesses a divergent filter, NSA/NPS/IVLL (39). TbAQP2 is a key drug transporter in T. brucei, mediating the uptake of pentamidine and melarsoprol, and its loss contributes to clinical drug resistance (35–37). In addition, TbAQP2 plays an important role in glycerol transport, as its loss increases parasite sensitivity to alternative oxidase inhibition, which leads to elevated intracellular glycerol levels (51). The *in vivo* roles of the other T. brucei aquaglyceroporins remain unknown, although all three are capable of arsenite and antimonite transport in yeast and Xenopus laevis heterologous expression systems (52). In contrast, our data demonstrate that in T. brucei, these transporters are selective for arsenic-containing melarsoprol (TbAQP2 [35]) and Sb(III) (TbAQP3). Intriguingly, RNAi library selection with SSG failed to identify TbAQP1, even though it contains the same selectivity filter as TbAQP3. This suggests important functional and regulatory differences between TbAQP1 and TbAQP3, which may influence their ability to contribute to Sb(III) uptake in bloodstream-form T. brucei. For example, TbAQP3 is localized to the plasma membrane in bloodstream-form T. brucei, and TbAQP1 localizes to the flagellar membrane (35, 53). This differential localization may influence their ability to mediate antimonial uptake.

The aminoglycoside paromomycin is thought to inhibit protein synthesis in *Leishmania* and enters the cell via endocytosis (21, 54, 55). However, RNAi library selection did not identify a surface receptor, suggesting that, at least in T. brucei, paromomycin entry is not dependent on a specific ligand-receptor interaction. Rather, the high endocytic flux associated with variant surface glycoprotein (VSG) internalization (56) may drive drug uptake. In addition, our screen did not identify proteins with identifiable roles in translational regulation. This perhaps surprising observation may be due to the relative essentiality of such proteins, whose loss, while capable of affecting paromomycin action, may also cause a substantial loss of fitness. Instead, RNAi fragments targeting Tb927.9.6360-80 dominated the paromomycin-selected RNAi library, with the remaining 28 high-confidence hits constituting only 9% of mapped reads. This locus encodes a set of closely related MFST proteins, at least one of which localizes to the lysosome, and has previously been associated with suramin efficacy (24). In contrast to paromomycin, several other endocytic pathway proteins, including three lysosomal proteins (p67, cathepsin L, and the MFST proteins), influence suramin efficacy (24). This led to the proposal that proteolytic processing in the lysosome releases suramin from bound proteins, enabling neutralization in the acidic environment or association with an alternative endogenous carrier and escape to the cytoplasm via one or more of the lysosomal MFSTs (57). In contrast, the absence of hits targeting other endocytic components following paromomycin RNAi library selection suggests little reliance on the endocytic network *per se*. Therefore, the lysosomal MFST proteins may influence paromomycin efficacy indirectly. MFST proteins mediate the transit of a diverse range of molecules, including polyamines and amino acids (41), and changes in the intracellular flux of these molecules may affect translation efficiency, which in turn may influence paromomycin efficacy. Deletion of the *Tb927.9.6360-80* locus from T. brucei yields only a 2-fold increase in the paromomycin EC_50_. However, the MFST protein encoded by the syntenic single-copy gene in *Leishmania* (e.g., *LmjF.15.0870*) remains to be characterized and may make a more substantial contribution to paromomycin action against this parasite.

Combination therapies are increasingly being used to treat leishmaniasis, enabling reduced dosing and treatment duration, resulting in fewer side effects (8). For example, a single dose of liposomal amphotericin B in combination with a short course of oral miltefosine or intramuscular paromomycin is an effective treatment for visceral leishmaniasis (VL) in the Indian subcontinent (58). In East Africa, SSG-paromomycin combination therapy is effective against VL (59). However, L. donovani parasites resistant to these and other antileishmanial drug combinations can be selected for *in vitro* (9, 10), and oxidative defense upregulation and changes in membrane fluidity have been associated with cross-resistance in laboratory-derived lines (23). Therefore, we carried out pairwise comparisons of our RNAi library screen data to identify potential cross-efficacy determinants. Only two hits fulfilled our stringency criteria, both of which influence amphotericin B and miltefosine action: TbVAMP7B, an endosomal SNARE protein responsible for endosome-lysosome fusion in other eukaryotes (45, 60), and Tb927.11.3350, the T. brucei orthologue of the *Leishmania* miltefosine transporter (17). However, while both of these proteins may influence membrane fluidity (see below), it seems unlikely that either one contributes significantly to oxidative defense. Recent Cos-seq gain-of-function analyses in L. infantum identified several candidate proteins whose overexpression reduces sensitivity to multidrug exposure (26); these proteins also lack an obvious connection to oxidative defense. Therefore, rather than being dependent on the increase or decrease in the expression of a single protein, changes in oxidative defense that lead to antileishmanial resistance are likely to be multifactorial. Our findings also suggest that amphotericin B-miltefosine combination therapy is most vulnerable to loss-of-function mutation, while others may be less susceptible to the downregulation of a single protein. This finding is particularly significant given that recent trials have confirmed the efficacy of amphotericin B-miltefosine combination therapy in treating VL (61, 62).

In contrast to the other antileishmanial drug efficacy determinants described here, TbVAMP7B depletion does not simply increase the drugs’ EC_50_s. Instead, TbVAMP7B RNAi knockdown reduces the amphotericin B EC_50_ and has little effect on the miltefosine EC_50_. The drop in the amphotericin B EC_50_ is due to a substantial decrease in the amphotericin B Hill coefficient, which has the opposite effect on EC_90_ and EC_99_, increasing both and enabling TbVAMP7B-depleted parasites to persist at these drug concentrations. Our data show that T. brucei has limited tolerance for TbVAMP7B depletion, presumably due to impairment of endosome-lysosome fusion (45). Intriguingly, exposure to low-concentration miltefosine complements the growth defect seen following TbVAMP7B depletion, suggesting that miltefosine treatment is able to promote vesicle membrane fusion in the endocytic system, a possible consequence of the enhanced membrane fluidity seen upon miltefosine exposure (63). TbVAMP7B has also recently been identified as a putative T. brucei apolipoprotein L1 (apoL1) sensitivity determinant (64), and other workers have highlighted the importance of the intracellular transit of the apoL1-carrying membrane for trypanolysis (65, 66). Our findings suggest that such transit also contributes to amphotericin B and miltefosine action. The VAMP7 proteins are highly conserved between T. brucei and *Leishmania* (43), suggesting that *Leishmania* parasites will also be sensitive to VAMP7B loss (LmjF.08.0030). However, subtle changes in VAMP7B expression that can be tolerated may enable parasites to take advantage of variations in amphotericin B and miltefosine tissue penetrance.

Miltefosine uptake in *Leishmania* is dependent on a phospholipid-transporting flippase (the MT) and its β-subunit, Ros3 (17, 18); both *in vitro*-selected lines and miltefosine-resistant L. donovani clinical isolates harbor mutations in the MT (7, 67, 68). Consistent with this, T. brucei RNAi library selection with miltefosine led to enrichment for RNAi fragments mapping to the syntenic sequence in T. brucei (*Tb927.11.3350*). RNAi library selection with amphotericin B also enriched for RNAi fragments mapping to this gene, consistent with recent findings in *Leishmania* (46), as well as two other flippases and a putative β-subunit (Tb927.11.13200). Interestingly, the β-subunit targeted was not the syntenic orthologue of Ros3, previously shown to interact with the MT (18). Therefore, different flippase/β-subunit dependencies may have evolved following the divergence of the *Leishmania* and T. brucei lineages. A further difference in the behaviors of these proteins between *Leishmania* and T. brucei lies in their localization. The MT and Ros3 localize to the plasma membrane in *Leishmania* (18), whereas in procyclic-form T. brucei, the MT orthologue (Tb927.11.3350) and a second flippase (Tb927.11.13000) localize to an intracellular structure reminiscent of the endosomal system (www.TrypTag.org) (69); their localization in BSF T. brucei is unknown. Therefore, while flippases influence drug action against *Leishmania* and T. brucei, they may mediate drug and/or phospholipid transit across different membranes in each parasite.

Phospholipid transport by flippases maintains the membrane asymmetry necessary for membrane fusion, vesicle trafficking, and sterol homeostasis (47). The identification of a single flippase following miltefosine selection is consistent with its role as a drug transporter (17). In contrast, amphotericin B selection identified three flippases, suggesting an indirect role in drug action, possibly through changes in membrane composition and transit through the endosomal system (Fig. 9). Amphotericin B acts by binding membrane ergosterol (70), leading to the formation of ion-permeable channels and downstream oxidative damage (71). Consistent with the importance of ergosterol to amphotericin B action, resistant clinical isolates exhibit elevated membrane fluidity and reduced ergosterol content (72). Recent findings have highlighted the mutation of key sterol biosynthetic enzymes, such as CYP51, and reduced ergosterol production as drivers of resistance in laboratory-derived amphotericin B-resistant Leishmania mexicana (73, 74). In contrast, amphotericin B selection of the BSF T. brucei RNAi library did not identify *Tb*CYP51 (Tb927.11.6210) or any other recognizable component of the ergosterol biosynthetic pathway, suggesting that limiting ergosterol production in BSF T. brucei is unable to reduce amphotericin B efficacy. However, our data indicate that changes in flippase expression may provide an alternative route to amphotericin B resistance. We speculate that reduced flippase activity may lead to changes in membrane ergosterol content or accessibility, thereby reducing the efficiency of amphotericin B binding and uptake. Therefore, functional characterization of the syntenic *Leishmania* orthologues of the T. brucei flippases may provide additional insights into the processes that drive the antileishmanial action of amphotericin B.

In summary, using our genome-scale BSF T. brucei RNAi library, we have identified a panel of putative antileishmanial drug efficacy determinants, highlighting two candidate cross-efficacy determinants, as well as roles for multiple flippases in the action of amphotericin B. The findings from this orthology-based chemogenomic profiling approach substantially advance our understanding of antileishmanial drug mode of action and potential resistance mechanisms and should facilitate the development of improved therapies as well as surveillance strategies to identify drug-resistant parasites.

## MATERIALS AND METHODS

### T. brucei strains.

MITat1.2/2T1 BSF T. brucei parasites (75) were maintained in HMI9 (50) (Invitrogen, LifeTech) supplemented with 10% fetal calf serum (Sigma) at 37°C with 5% CO_2_. Transfection was carried out in either cytomix or Tb-BSF buffer (76), for integration at the 2T1 “landing pad” (75, 77) or *Tb927.9.6360-80*, respectively, using a Nucleofector (Lonza) set to program X-001. Transformants were selected with 2.5 μg · ml^−1^ hygromycin, 2 μg · ml^−1^ puromycin, or 10 μg · ml^−1^ blasticidin, as appropriate. The BSF T. brucei RNAi library was maintained with 1 μg · ml^−1^ phleomycin and 5 μg · ml^−1^ blasticidin (34). For growth assays, cultured BSF T. brucei parasites were seeded at ∼10^5^ cells · ml^−1^, counted using a hemocytometer, and diluted back every 24 h, as necessary, for 3 days in the absence of antibiotics. All selective antibiotics were purchased from InvivoGen.

### Drug sensitivity assays.

Half-maximal effective concentrations (EC_50_s) of the antileishmanial and antitrypanosomal drugs (sodium stibogluconate [GSK], paromomycin [Sigma], miltefosine [Paladin], amphotericin B [E. R. Squibb, UK], pentamidine [Sigma], and suramin [TDR/WHO]) and neomycin (G418; InvivoGen) were determined over 78 or 30 h. BSF T. brucei parasites were seeded at 2 × 10^3^ cells · ml^−1^ (or 2 × 10^5^ cells · ml^−1^) in 96-well plates with a 2-fold dilution series of each drug; assays were carried out in the absence of other antibiotics. After 72 or 24 h, resazurin (Sigma) in phosphate-buffered saline (PBS) was added to a final concentration of 12.5 μg · ml^−1^ per well, and the plates were incubated for a further 6 h at 37°C. Fluorescence was determined using a fluorescence plate reader (Molecular Devices) at an excitation wavelength of 530 nm, an emission wavelength of 585 nm, and a filter cutoff of 570 nm (78). Data were processed in Microsoft Excel, and nonlinear regression analysis was carried out with GraphPad Prism. The short-term kinetics of killing at high concentrations of the drug (>EC_99_) were determined in triplicate over 24 h from a starting cell density of 1 × 10^5^ cells · ml^−1^.

### T. brucei RNAi library screening and RIT-seq.

RNA library screening was carried out as previously described (34). Briefly, library expression was induced with 1 μg · ml^−1^ tetracycline (Sigma) for 24 h prior to selection with each antileishmanial drug at 1× to 3× EC_50_. Cell density was assessed daily using a hemocytometer and diluted to no less than 20 million cells in 100 ml medium; induction and antileishmanial drug selection were maintained throughout. Once robust growth had been achieved for at least 2 days, genomic DNA was prepared for RNAi target identification. The RNAi cassettes remaining in the antileishmanial-selected RNAi libraries were amplified from genomic DNA using the primer pair LIB2F/LIB2R and sequenced on an Illumina HiSeq platform at the Beijing Genome Institute.

The sequenced RNAi target fragments were mapped against the T. brucei strain TREU927 reference genome (release 6.0), as described previously (34). Briefly, mapping was carried out using Bowtie2 (79) set to “very sensitive local” alignment, and output SAM files were processed using SAMtools (80). The resultant BAM files were viewed against the reference genome in the Artemis genome browser (81). Reads containing the RNAi construct-specific 14-base barcode were identified using a custom script (34) and corresponded to at least 22% of reads from each selected RNAi library. This subset of reads was mapped against the TREU927 reference genome, as described above. Plots were generated using the Artemis graph tool and processed in Adobe Photoshop Elements 8.0. Stacks of reads that included the 14-base barcode on the positive strand were used to define RNAi target fragment junctions and to assign high-confidence hits as those identified by at least two RNAi target fragments. RNAi target fragment read numbers were converted to RPKM (reads per kilobase per million reads mapped) to account for interlibrary read depth variations when comparing RNAi library sequencing outputs.

Alignments were carried out in Clustal Omega (https://www.ebi.ac.uk/Tools/msa/clustalo/), unrooted neighbor-joining trees were formatted in Dendroscope 3 (http://dendroscope.org/) (82), and putative *trans*-membrane domains were identified using TOPCONS (http://topcons.cbr.su.se/) (83). GO term profiles were constructed using the GO analysis tool at http://tritrypdb.org.

### Plasmid and T. brucei strain construction and analysis.

*Tb927.9.6360-80* locus deletion constructs were assembled by incorporating targeting fragments flanking either a puromycin acetyltransferase (PAC) or a blasticidin S deaminase (BSD) open reading frame. Deletion constructs were linearized with NotI/ApaI (New England Biolabs [NEB]) to expose the flanking targeting fragments prior to transfection. Stem-loop RNAi constructs targeting Tb927.11.6680 (AAT15), Tb927.11.13000, Tb927.11.3350, Tb927.5.3560 (TbVAMP7B), and Tb927.5.3570 were assembled in pRPa-iSL (77). RNAi targeting fragments were designed using the RNAit primer design algorithm to minimize off-target effects (84). pRPa-iSL constructs were linearized with AscI (NEB) prior to transfection and targeted integration at the ribosomal DNA (rDNA) spacer landing-pad locus in 2T1 BSF T. brucei (75). Details of all primers are available upon request. Tb927.9.6360-80 allelic replacement was confirmed by Southern hybridization following XhoI (New England Biolabs) digestion of genomic DNA. RNAi knockdown was confirmed by northern hybridization of total RNA or, in the case of Tb927.11.6680, by reverse transcription-quantitative PCR (RT-qPCR), as described previously (85). For Southern and Northern hybridization, digoxigenin-dUTP (Roche)-labeled DNA probes were generated by PCR, hybridized, and detected according to standard protocols and the manufacturer’s instructions.

### Data availability.

Sequence data are available as FASTQ files at the European Nucleotide Archive (https://www.ebi.ac.uk/ena) under study accession number PRJEB31973 (amphotericin B, accession number ERS3348616; miltefosine, accession number ERS3348617; paromomycin, accession number ERS3348618; sodium stibogluconate, accession number ERS3348619).

## Supplementary Material

Supplemental file 1

Supplemental file 2

Supplemental file 3
