# Supplementary material for: Chemogenomic Profiling of Antileishmanial Efficacy and Resistance in the Related Kinetoplastid Parasite Trypanosoma brucei
Source: Antimicrob Agents Chemother. 2019 Jul 25;63(8):e00795-19. doi: 10.1128/AAC.00795-19 (PMC6658743; doi:10.1128/AAC.00795-19)
Supplement: Supplemental file 1 [file AAC.00795-19-s0001.pdf]

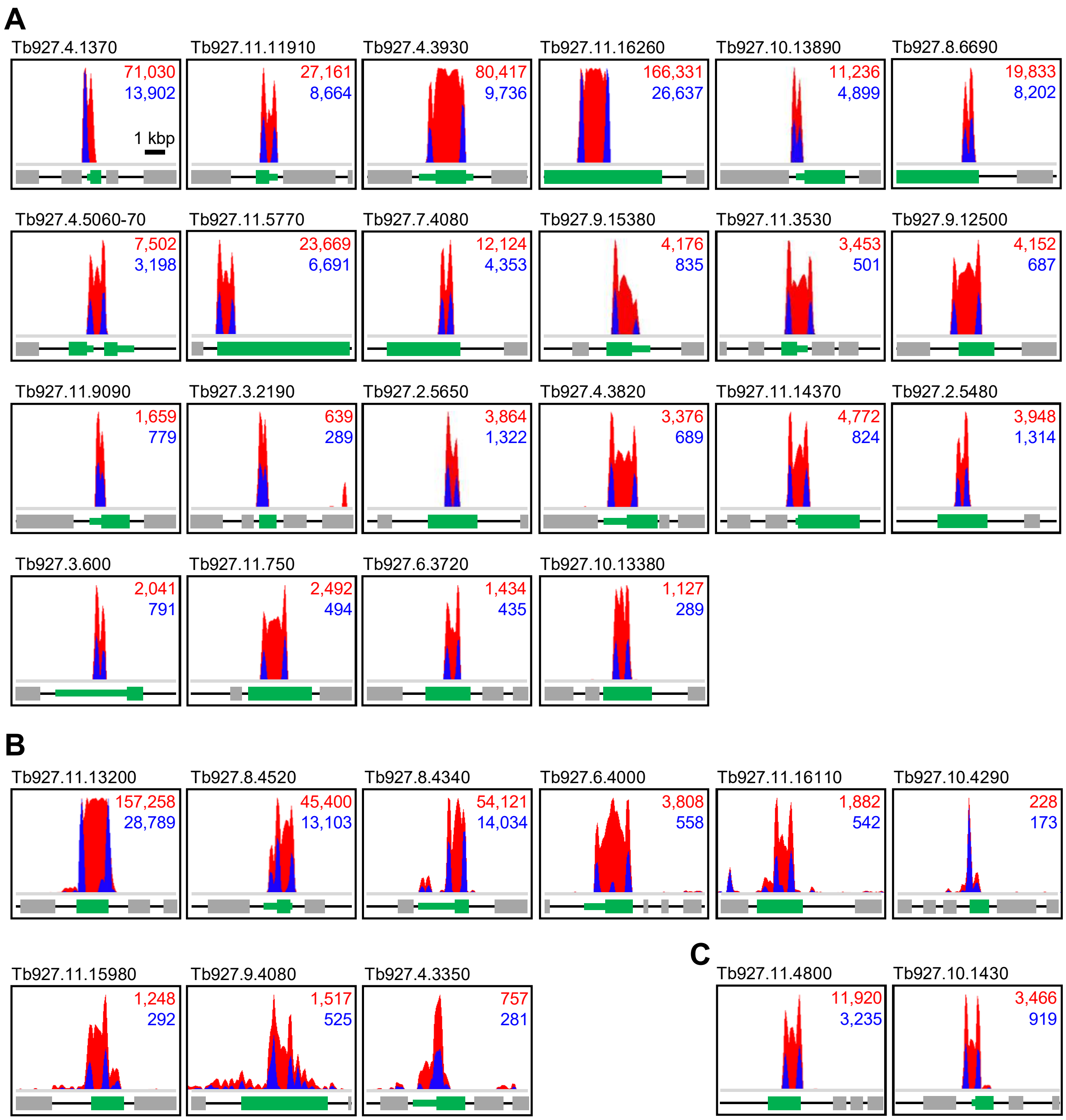

Figure S1: Candidate anti-leishmanial drug efficacy determinants identified by *T. brucei* RNAi library selection. Total (red) and RNAi construct-specific 14mer-containing (blue) reads mapping to individual loci following BSF *T. brucei* RNA library selection in paromomycin (A), amphotericin-B (B) and miltefosine (C). Targeted open reading frames highlighted in green; flanking open reading frames coloured grey. Where a substantial number of reads target regions outside the open reading frame, the predicted untranslated region is highlighted by a narrow green bar. See Table S1 for further details.

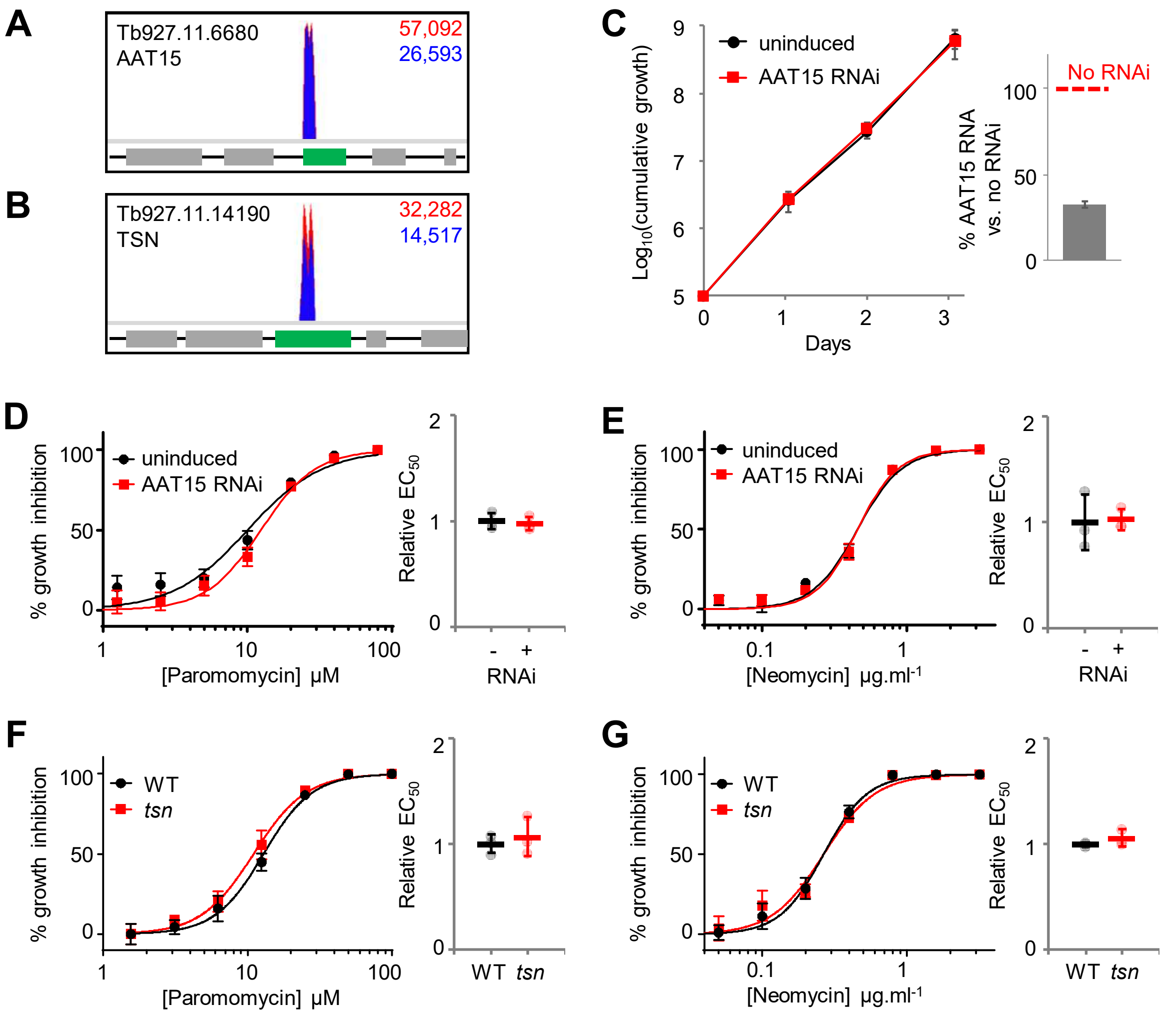

Figure S2: Neither AAT15 (Tb927.11.6680) depletion nor Tudor Staphylococcal nuclease (Tb927.11.14190) deletion affects aminoglycoside efficacy against BSF *T. brucei* over 72 hours. A, B) Total (red) and RNAi construct-specific 14mer-containing (blue) reads mapping to *Tb927.11.6680* (A) and *Tb927.11.14190* (B) following paromomycin selection. Targeted open reading frames highlighted in green; flanking open reading frames coloured grey. C) *T. brucei* population growth following AAT15 RNAi knockdown. Inset: RNA depletion was confirmed by RT-qPCR following 24-hour induction in 1 $\mu g.ml^{-1}$ tetracycline. D, E) Representative paromomycin and neomycin EC_{50} assays following AAT15 RNAi knockdown induced in 1 $\mu g.ml^{-1}$ tetracycline. F, G) Representative paromomycin (D) and neomycin (E) EC_{50} assays comparing wild type and *Tb927.11.14190* null (*tsn*) BSF *T. brucei*. Inset charts summarise data from three independent biological replicates. Individual growth (C) and EC_{50} (D-G) assays were carried out in triplicate and quadruplicate, respectively. Error bars represent standard deviation.

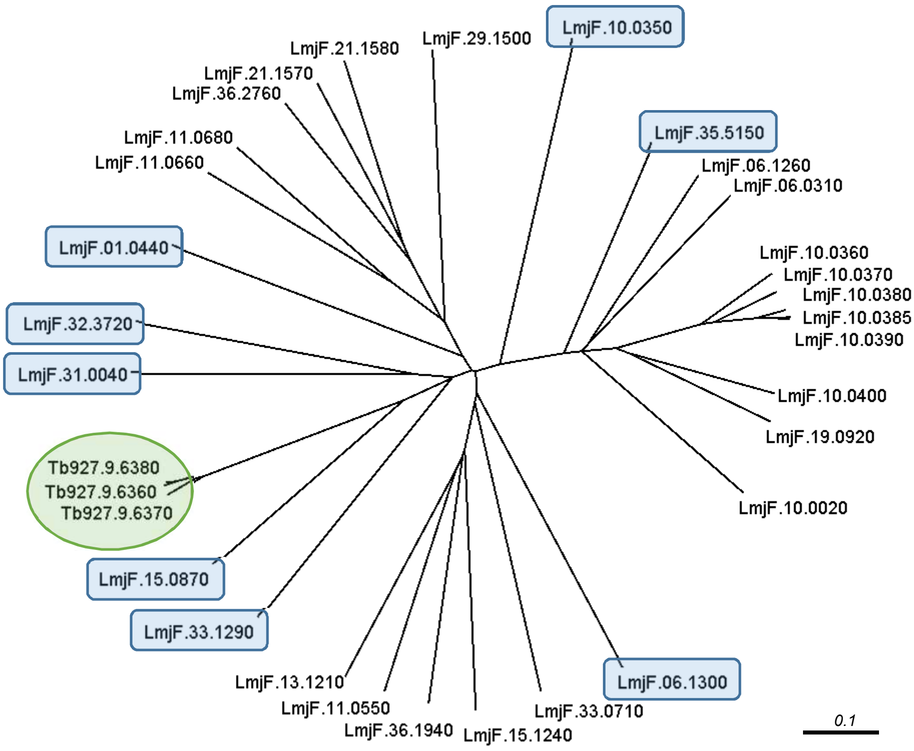

Figure S3: *Tb927.9.6360-80* clusters with the syntenic *LmjF.15.0870*. Twenty nine open reading frames annotated 'major facilitator' or 'MFS' in the *L. major* Friedlin reference genome were aligned with the Tb927.9.6360-80 open reading frames using Clustal Omega (https://www.ebi.ac.uk/Tools/msa/clustalo/). The unrooted neighbour joining phylogenetic tree was formatted in Dendroscope 3 (http://dendroscope.org/).

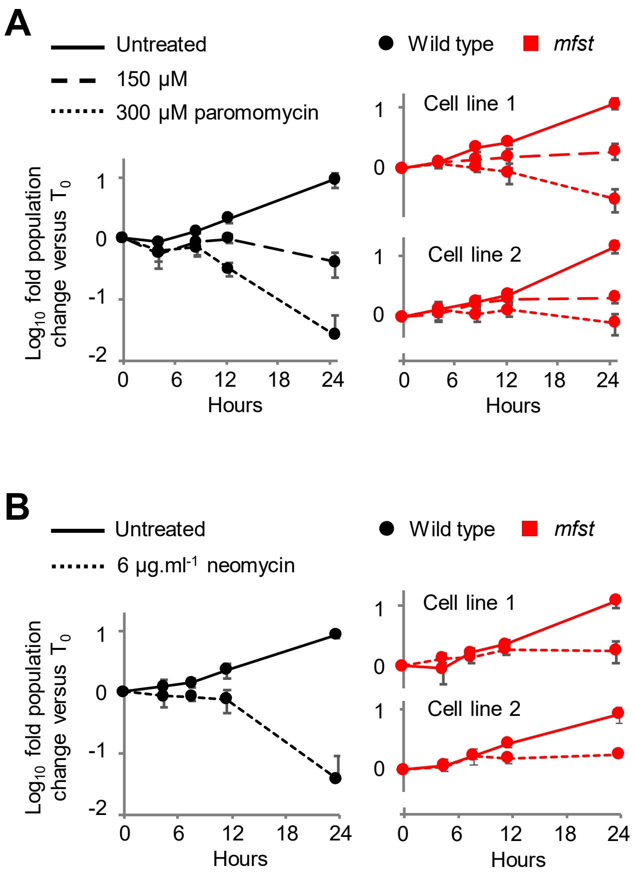

Figure S4: *MFST* locus null *T. brucei* exhibit enhanced tolerance to high concentration aminoglycosides. Relative population growth of wild type (WT) and *MFST* locus null (*mfst*) *T. brucei* in A) paromomycin and B) neomycin at >EC_{99}. Assays were carried out in triplicate. Error bars represent standard deviation.

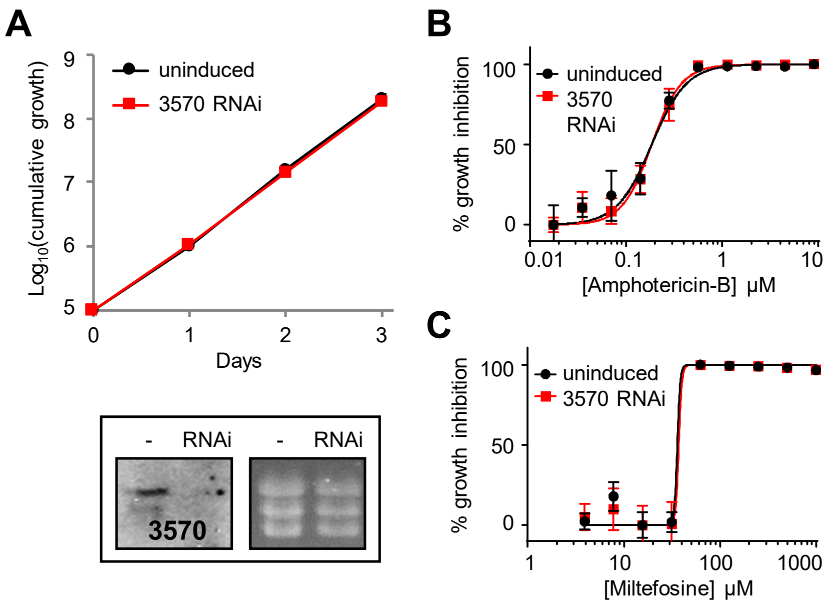

Figure S5: Tb927.5.3570 does not contribute to the efficacy of amphotericin-B or miltefosine against *T. brucei*. A) *T. brucei* population growth following Tb927.5.3570 RNAi knockdown. Inset: confirmation of RNAi knockdown by northern blot; ethidium bromide stained gel shown as a loading control. B, C) Representative amphotericin-B and miltefosine EC_{50} assays following Tb927.5.3570 RNAi knockdown. RNAi inductions were carried out in 1 μg.ml^{-1} tetracycline. Individual growth (A) and EC_{50} (B, C) assays were carried out in triplicate and quadruplicate, respectively. Error bars represent standard deviation.

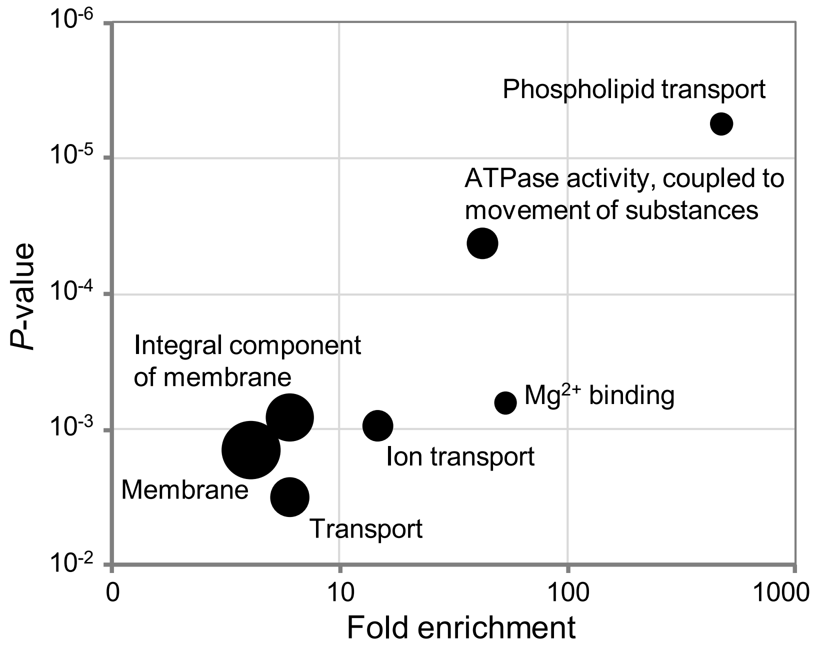

Figure S6: Gene Ontology analysis of the high confidence hits identified following amphotericin-B RNAi library selection. Plot generated using the GO analysis tool at TritrypDB.org. Point diameter corresponds to relative number of proteins in each category. See Table S2 for further details.

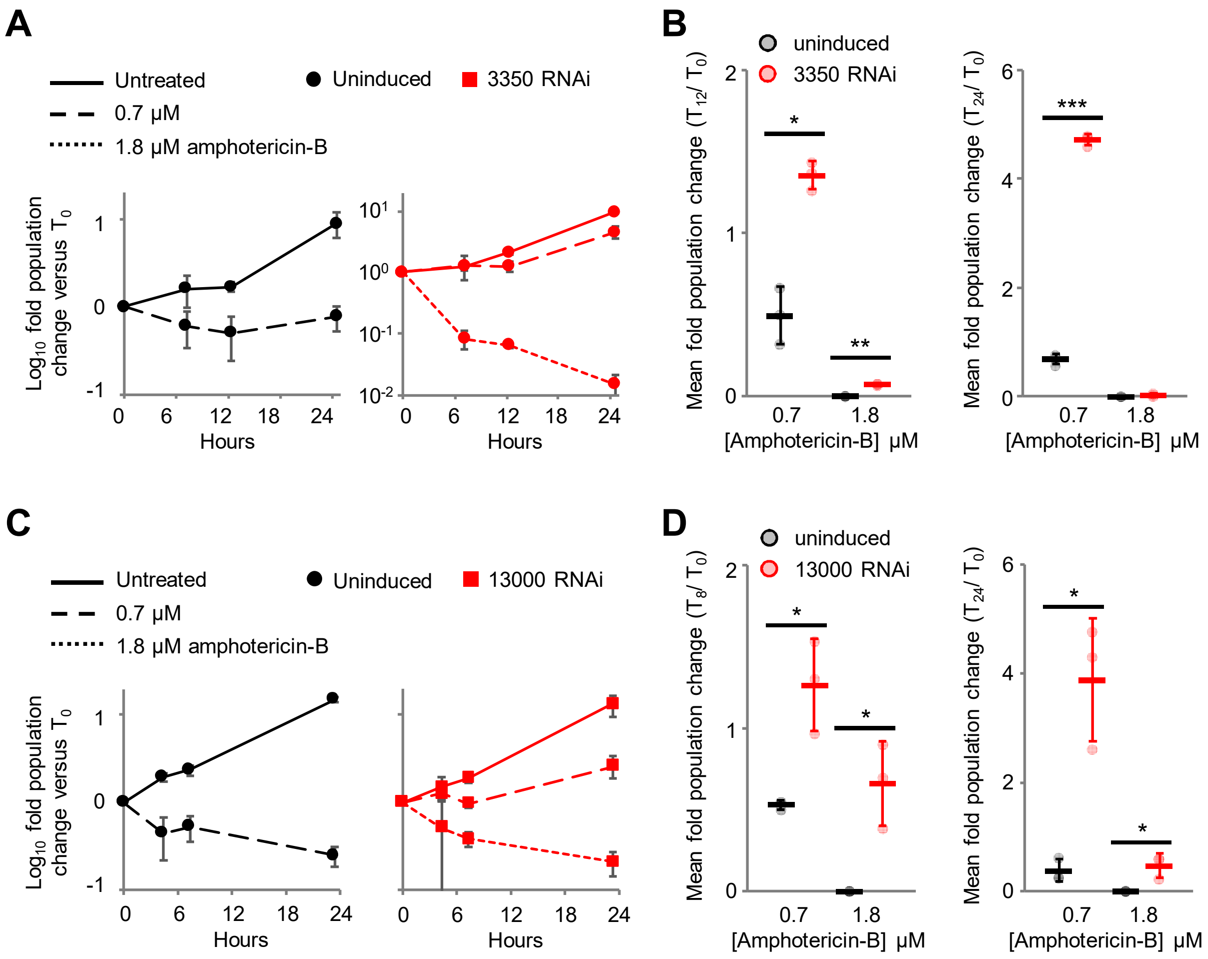

Figure S7: *T. brucei* exhibit enhanced tolerance to high concentration amphotericin-B following flippase depletion. A, C) Representative assays showing relative population growth in >EC_{99} amphotericin-B following (A) Tb927.11.3350 and (C) Tb927.11.13000 RNAi knockdown. B, D) Relative population growth in >EC_{99} amphotericin-B following (B) Tb927.11.3350 and (D) Tb927.11.13000 RNAi knockdown; data derived from three independent biological replicates. Individual growth assays were carried out in triplicate. Error bars represent standard deviation. *P*-values derived from Student's *t*-test (* <0.05; *** <0.001). RNAi inductions were carried out in 1 μg.ml^{-1} tetracycline.
